# Survival trends for small intestinal cancer in England and Wales, 1971–1990: national population-based study

**DOI:** 10.1038/sj.bjc.6603417

**Published:** 2006-10-10

**Authors:** N Pashayan, C Lepage, B Rachet, L M Woods, M P Coleman

**Affiliations:** 1 Department of Public Health and Primary Care, Institute of Public Health, University Forvie Site, Robinson Way, Cambridge CB2 2SR, UK; 2 Non-communicable Disease Epidemiology Unit, London School of Hygiene and Tropical Medicine, Keppel Street, London WC1E 7HT, UK

**Keywords:** small intestinal cancers, relative survival, trends, prognostic factors, population-based

## Abstract

This population-based study examines prognostic factors and survival trends among adults (15–99 years) diagnosed with small intestinal cancer in England and Wales during 1971–1990 and followed up to 1995. During this period, the 1- and 5-year age-standardised relative survival rates for small intestinal cancers combined were 42% and 23%, respectively. Duodenal tumours, adenocarcinomas, men, patients with advanced age and the most deprived patients had the poorest prognosis. For all small bowel tumours combined, the excess risk of death fell significantly by 6–9% every 4 years over the 20-year period (adjusted excess hazard ratio (EHR) 0.91 at 1 year after diagnosis, 0.94 at 5 years). For duodenal tumours, the EHR fell by about 14% (95% CI 5–22%) every 4 years between 1979 and 1990, and a similar trend for jejunal tumours was of borderline significance. Further population-based investigations linking survival data to individual data on diagnostic methods and types of treatment are needed.

The small intestine constitutes 75% of the length of the alimentary tract, but accounts for only 1–2% of gastrointestinal cancers (Wiggers, 2002). The duodenum, jejunum and ileum vary in function and in the biochemical composition of food end products that flow through them (Chow *et al*, 1996). Small intestinal cancers are heterogeneous: the predominant morphologic type varies with the anatomic site, and cancers of the same type behave differently at different sites.

Few population-based studies of small intestinal malignancy exist, and what is known of its survival derives mostly from small hospital case series. No consensus exists on whether survival varies with the anatomic origin of the tumour (Zar *et al*, 1996; Howe *et al*, 1999; Howe *et al*, 2001). In England and Wales, survival from small intestinal cancer in adults improved between 1971 and 1995 (Coleman *et al*, 1999). These trends may be due to change in the relative frequency of cancers with better or worse prognosis, or availability of and accessibility to more effective diagnostic methods and treatment, and/or diagnosis at an earlier and more treatable stage.

This population-based study was designed to describe the epidemiology of small intestinal cancer, to examine survival trends and to explore the impact of prognostic factors on trends in excess mortality from tumours arising in different parts of the small bowel.

## MATERIALS AND METHODS

### Data

A detailed description has been published (Coleman *et al*, 1999). Briefly, as part of the national cancer registration scheme (OPCS, 1990), the Office for National Statistics (ONS) compiled data on individual cancer patients in the National Cancer Registry (NCR) from 12 regional cancer registries in England and Wales. Linkage to the National Health Service Central Register (NHSCR), a virtually complete national person index, provides the NCR with notification of the eventual death or emigration of any patient with cancer whose record is flagged at NHSCR.

We extracted data for all patients diagnosed aged 15–99 years with a primary malignancy of the small bowel in England and Wales between 1 January 1971 and 31 December 1990 and followed up to the end of 1995. After the exclusion of 925 patients, mainly because of multiple/synchronous tumours (30 patients), unknown survival (591), age below 15 or above 99 (eight) or unknown vital status (296), 5612 subjects (86% of those eligible) were included in the analyses. Patients with lymphoma of the small intestine were not included in this study.

Information available for each patient included sex, date of birth, date of diagnosis, morphological and anatomic site codes, vital status and date of death, NHS Region and deprivation category. The Carstairs index was used as a measure of deprivation (OPCS, 1990), estimated at the level of the census enumeration district (ED, mean population 500). A deprivation category was assigned on the basis of the patient's ED of residence at diagnosis, using contemporary dictionaries of correspondence between postcode of residence and ED.

Tumours located in the jejunum only became separately identifiable from other small bowel tumours from 1979, with the introduction of the ninth edition of the International Classification of Diseases (ICD-9) (World Health Organization, 1977), so the data were examined in five 4-year periods of diagnosis (1971–1974, 1975–1978, 1979–1982, 1983–1986 and 1987–1990). Patients were censored alive from analysis on the date of emigration or 31 December 1995, whichever was earlier.

Trends in the distribution of cases by tumour and patient factors were examined with a score test for linear trend. Relative survival up to 5 years was estimated for England and Wales by age, sex, anatomic localisation, morphologic type and calendar period of diagnosis, using a maximum likelihood method for individual tumour records (Estève *et al*, 1990). Relative survival reflects the excess mortality in the cancer patient group relative to the expected or background mortality in the general population. It can be considered as survival in the absence of other causes of death (competing mortality), which itself varies widely between population subgroups. The life tables used to represent the expected mortality incorporated death rates for England and Wales by single year of age at death (15–99), sex and every combination of deprivation category, NHS Region and calendar period (1971–1985, 1986–1995) (Coleman *et al*, 1999). National estimates of relative survival do incorporate regional differences in background mortality, but results are not presented here by NHS Region.

Relative survival estimates at 1 and 5 years after diagnosis were age-standardised, with weights given by the distribution across five age groups (15–49, 50–59, 60–69, 70–79 and 80–99 years) of adults diagnosed with small intestinal cancer during 1987–1990. The choice of standard weights is arbitrary, but the age-standardised estimates are not widely different from the raw estimates, and they enable direct comparison of relative survival over time, compensated for changes in the age distribution of patients.

The simultaneous effects of covariates on relative survival were estimated with regression models as the excess hazard ratio (EHR) (Dickman *et al*, 2003). Follow-up time was divided into yearly intervals, and models were estimated both for the first year and up to the fifth anniversary of diagnosis. The significance of main effects, linear trends and interactions between follow-up time and other covariates was assessed by likelihood ratio tests at the 5% level. A forward-fitting strategy was used to select variables that contributed significantly to the final model. Analyses were done in STATA® 7.0 (Stata Corp, 2001).

## RESULTS

### Characteristics

Almost two-thirds (58%) of cases arose in people aged 60–79 years (Table 1). There was little difference between men and women. During 1987–1990, duodenum was the most common subsite (29%), followed by ileum (21%) and jejunum (10%), but for more than a third of tumours, the subsite was not specified. During 1987–1990, half of all small bowel tumours were adenocarcinomas (51%), but this varied widely with subsite, from jejunum (67%) to ileum (37%) (*P*<0.001). Similarly, endocrine tumours comprised 14% of all tumours, varying widely from ileum (33%) to duodenum and jejunum (2%).

### Relative survival

Age-standardised relative survival for all small intestinal tumours combined was 42% at 1 year, 28% at 5 years and 19% at 10 years. Among tumours of specified subsite, duodenal tumours had the lowest survival (29% at 1 year, 13% at 5 years) and ileal tumours the highest (56%; 34%). Among tumours of specified morphology, endocrine tumours had the highest survival (66% at 1 year, 46% at 5 years). Relative survival for duodenal adenocarcinomas was 42% (standard error (s.e.) 1.7%) at 1 year and 15% (s.e. 1.3%) at 5 years, significantly lower than for adenocarcinomas arising either in the jejunum (60% (s.e. 2.8%) and 26% (s.e. 2.7%)) or in the ileum (57% (s.e. 2.7%) and 25% (s.e. 2.5%)).

**Figure 1:**
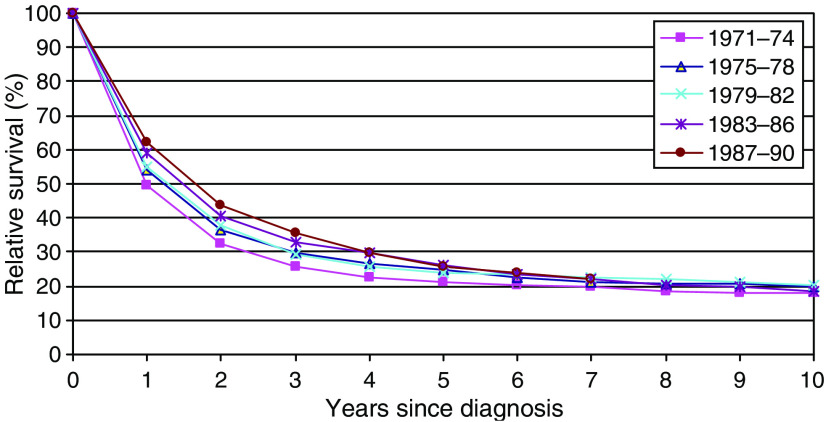
Age-standardised relative survival for small intestinal tumours, adults (15–99 years) diagnosed in England Wales 1971–1990, followed up to 1995: by period of diagnosis.

Over the 20-year period 1971–1990, the mean absolute increase in age-adjusted relative survival every 4 years was 3.1% at 1 year after diagnosis (*P*<0.001) and 1.1% at 5 years (*P*=0.009), but only 0.5% at 10 years (*P*=0.421; Figure 1).

### Prognostic factors

Compared to patients with duodenal tumours (reference category), those with ileal and jejunal tumours had a 35–40% lower excess risk of death within the first 5 years (EHRs 0.60–0.65, adjusted for all other covariates; Table 2). Patients with endocrine tumours only had about half the excess mortality (EHR 0.48) of those with adenocarcinoma (reference group). Women had 8–13% lower excess mortality than men. The excess risk of death in the first year after diagnosis was 33% higher among the most deprived than the most affluent patients (adjusted EHR 1.33, 95% CI 1.17–1.52), and the excess hazard was still 22% higher over the first 5 years after diagnosis, with a clear gradient across socio-economic groups (*P*-trend <0.001).

### Trends in the excess risk of death

Overall, there was a significant 6–9% fall every 4 years in the excess risk of death from small bowel tumours, both at 1 year after diagnosis (EHR 0.91) and at 5 years (EHR 0.94; Table 2). Compared with patients diagnosed during 1971–1974, the adjusted excess risk of death for those diagnosed during 1987–1990 had fallen to 0.65 at 1 year after diagnosis and to 0.74 at 5 years.

Time trends in the excess hazard of death from tumours in each part of the small bowel were less clear-cut. For duodenal tumours, the excess risk of death at 1 year fell significantly by 26% (EHR 0.74) in the 8 years between 1979–1982 and 1987–1990, after adjustment for age, deprivation and morphology (average fall 14% every 4 years, EHR 0.86, *P*-trend=0.004), and the excess hazard at 5 years also fell by about 10% every 4 years (Table 3). The excess risk of death for jejunal tumours fell at a similar rate, but the numbers of patients and deaths were smaller than for duodenal tumours, and this trend was of borderline significance (*P*-trend=0.090). The excess risk of death at 1 year also fell for ileal tumours, but the trend was not statistically significant.

## DISCUSSION

To our knowledge this is the first population-based study to examine survival from small intestinal tumours separately by morphology, subsite and socio-economic deprivation. The large number of cases from a long-standing national cancer registry with up to 25 years of follow-up also enabled analysis of survival trends for these rare tumours.

The anatomic origin of the tumour is a significant prognostic factor. Ileal tumours had the highest survival, followed by those of the jejunum and duodenum. Survival has not been shown to vary with subsite in small case series from single institutions (Zollinger, 1986; Cunningham *et al*, 1997; Ojha *et al*, 2000). Other studies (Ashley and Wells, 1988; Frost *et al*, 1994) have shown slightly more favourable prognosis for duodenal adenocarcinomas. However, hospital-based series are prone to selection bias. Studies in the USA, based on hospital data provided voluntarily to the National Cancer Patient Data Base, showed that adenocarcinomas (Howe *et al*, 1999) and sarcomas (Howe *et al*, 2001) of the duodenum had significantly lower survival than those in the jejunum and ileum. A population-based study using Swedish Cancer Registry data also showed that adenocarcinomas of the duodenum had the worst prognosis (Zar *et al*, 1996).

Lower survival for duodenal tumours is clinically plausible. Surgical management may involve duodenopancreatectomy, which is associated with much higher mortality and morbidity than resection of jejunal or ileal tumours (Al-Sharaf *et al*, 1999), and vascular invasion often limits surgery. Not all patients with duodenal tumours would be resectable, and surgery is the only curative treatment. In this study, the proportion of patients with duodenal tumours aged 70 years or over was higher than for jejunal or ileal malignancies. Howe *et al* (1999) found that only about half of duodenal tumours were treated by surgery of curative intent, compared with 90% for jejunal and ileal tumours.

Improvement in survival from small intestinal tumours has been reported from Sweden by Zar *et al* (1996) in their population-based study. The relative hazard of death from adenocarcinoma of the small intestine in the late 1980s was significantly lower than for 1960–1970. The improvement was confined to duodenal adenocarcinomas and did not affect tumours of the jejunum or ileum. Here, we found similar improvements in survival for duodenal, jejunal and ileal tumours, but the trend was statistically significant only for duodenal tumours.

Several explanations are possible for the overall improvement in survival for small intestinal tumours in England and Wales. Survival for most cancers diagnosed during 1971–1990 has improved steadily (Coleman *et al*, 1999). This can be attributed to improvement in general cancer care, including improvement in imaging techniques (Ullerich *et al*, 2001; Beal *et al*, 2002; de Mascarenhas-Saraiva and da Silva Araùjo Lopes, 2003). Increased use of faecal occult blood tests and increased awareness of symptoms may also have contributed to earlier diagnosis. Biologic and environmental factors influencing expression and behaviour of the tumours cannot be excluded (Adami *et al*, 1989).

Most of the improvement was seen in the first year after diagnosis, with more modest gains in 5- and 10-year survival. No major new treatment modalities have been introduced (Zar *et al*, 1996) and chemotherapy has not been systematically evaluated (Copson and Iveson, 2003), so this pattern of improvement in survival suggests that perioperative complications have become less common, and the proportion of patients who are effectively cured of their malignancy has not improved.

A major advantage of this study is the population-based data on more than 5600 patients with small intestinal cancer diagnosed in England and Wales over the 20-year period 1971–1990. All data were collected using multiple sources of information in a uniform manner, regardless of the time period and centre of diagnosis. Nearly all persons newly diagnosed with a small intestinal tumour in a population of over 50 million will have been registered, avoiding the selection bias that is common in hospital case series.

This study has several limitations. Incidence and survival trends for jejunal and ileal tumours could only be studied from 1979, when they were first distinguished from other intestinal tumours in the ICD. Stage of disease is an important prognostic factor but it was not available in the national data. The use of ecological measures of deprivation was imposed because no individual measures are available, but this could only have attenuated the significant association we observed between deprivation and survival. Evidence is also available that small-area indicators of deprivation do reflect individual disadvantage in relation to health (Sloggett and Joshi, 1998).

In conclusion, survival from small intestinal malignancy in England and Wales has improved, possibly owing to earlier diagnosis and better treatment. Survival varies widely by subsite within the small bowel and by morphologic type. Further studies are needed to assess the impact of endoscopic examination on early detection and improved survival. Linkage of population-based cancer registry data with detailed clinical data on diagnosis, pathology and treatment would improve the explanatory power of studies such as this: the results could better inform healthcare planners about sustainable measures for improved survival.

## Figures and Tables

**Table 1:** Small intestinal cancer: number of patients and distribution (%) of tumour and patient characteristics by period of diagnosis: England and Wales adults (15–99 years) diagnosed 1971–1990

	**Period of diagnosis**					**All**	
	**1971–1974**	**1975–1978**	**1979–1982**	**1983–1986**	**1987–1990**	**periods**	***P*-trend** a
No. of patients	1012	1091	1107	1200	1202	5612	
*Tumour characteristics*							
*Subsite*							
Duodenum	24.3	26.1	25.3	25.6	28.9	26.1	0.034
Jejunum	—b	—b	13.6	12.0	10.1	7.4	0.008^c^
Ileum	—b	—b	25.3	24.8	21.4	14.9	0.026^c^
Other	44.4	41.3	3.3	1.7	1.5	17.4	0.002^c^
Unspecified	31.3	32.5	32.4	35.9	38.1	34.2	<0.001
							
*Morphology*							
Adenocarcinoma	41.4	41.2	45.8	48.3	50.5	45.7	<0.001
Endocrine tumour	13.0	13.4	15.1	16.6	13.5	14.4	0.247
Sarcoma	12.5	9.3	10.0	10.7	7.5	9.9	0.003
Other	33.1	36.1	29.1	24.4	28.5	30.0	<0.001
							
*Patient characteristics*							
*Sex*							
Male	53.6	53.6	49.3	50.1	49.3	50.5	0.06
Female	46.4	46.4	50.7	49.9	50.7	49.5	0.06
							
*Age at diagnosis (years)*							
15–49	11.4	13.2	9.5	10.6	10.9	11.1	0.236
50–59	20.0	20.2	18.3	17.1	16.5	18.3	0.006
60–69	32.2	27.4	31.6	28.1	29.2	29.6	0.237
70–79	25.5	28.9	28.2	32.1	27.9	28.6	0.074
80–99	10.9	10.3	12.4	12.1	15.5	12.4	<0.001
							
*Deprivation*^d^							
Affluent	18.3	17.7	19.9	19.9	21.9	19.7	0.016
2	21.1	24.4	21.3	22.2	20.5	21.9	0.296
3	22.7	21.2	21.8	21.3	21.9	21.7	0.806
4	20.3	20.4	19.5	20.2	19.1	19.9	0.525
Deprived	17.6	16.3	17.5	16.4	16.6	16.8	0.653

a *P*-value derived from score test for trend.

b No data – jejunum and ileum were separately identified only from 1979, in ICD-9 (see text).

c Applies for periods 1979–1990.

d Total *N*=5237, but 375 (7%) cases could not be assigned to a deprivation category (see text).

**Table 2:** Adjusted excess hazard ratio (EHR) and 95% confidence intervals (CI) at 1 year and 5 years after diagnosis, by prognostic factor: small intestinal malignancy, England and Wales, adults diagnosed 1971–1990 and followed up to 1995

	**Adjusted excess hazard ratio (EHR)^a^**			
	**1 year**		**5 years**	
**Period of diagnosis**	**EHR**	**95% CI**	**EHR**	**95% CI**
1971–1974	1.00	—	1.00	—
1975–1978	0.87	0.77–0.99	0.87	0.80–1.00
1979–1982	0.84	0.74–0.96	0.89	0.83–1.03
1983–1986	0.82	0.72–0.93	0.86	0.80–0.99
1987–1990	0.65	0.57–0.74	0.74	0.69–0.86
Trend^b^	0.91	0.89–0.94	0.94	0.92–0.96
				
*Subsite*				
Duodenum	1.00	—	1.00	—
Jejunum	0.59	0.50–0.71	0.65	0.57–0.75
Ileum	0.62	0.53–0.72	0.65	0.57–0.73
Other	1.11	0.81–1.54	1.12	0.86–1.47
Unspecified	0.86	0.76–0.97	0.78	0.70–0.86
				
*Morphology*				
Adenocarcinoma	1.00	—	1.00	—
Endocrine tumour	0.48	0.41–0.56	0.48	0.42–0.54
Sarcoma	0.72	0.61–0.83	0.91	0.81–1.02
Other	1.92	1.76–2.09	1.61	1.49–1.73
				
*Sex*				
Male	1.00	—	1.00	—
Female	0.87	0.81–0.94	0.92	0.86–0.98
				
*Age at diagnosis (years)*				
15–59	1.00	—	1.00	—
60–69	1.22	1.10–1.36	1.19	1.09–1.29
70–79	1.58	1.43–1.76	1.42	1.31–1.55
80–99	2.46	2.16–2.79	2.13	1.91–2.39
				
*Deprivation*				
Affluent	1.00	—	1.00	—
2	1.01	0.89–1.14	0.97	0.88–1.07
3	1.04	0.92–1.17	0.97	0.88–1.08
4	1.24	1.09–1.40	1.11	1.00–1.23
Deprived	1.33	1.17–1.52	1.22	1.09–1.36

a Adjusted for each covariate in the table by stepwise addition, with likelihood ratio test of the significance of the variable on the goodness of fit of the model.

b Ratio of the excess hazard of death between successive calendar periods of diagnosis, after adjustment for other covariates in the Table.

**Table 3:** Adjusted excess hazard ratios (EHR) and 95% confidence intervals (CI) for short-term and longer-term follow-up, by period of diagnosis and subsite: England and Wales, adults diagnosed with small intestinal cancer 1979–1990 and followed up to 1995

**Duration of follow-up**	**Duodenum^a^**		**Jejunum^b^**		**Ileum^c^**		**Unspecified site^d^**	
**Period of diagnosis**	**EHR**	**95% CI**	**EHR**	**95% CI**	**EHR**	**95% CI**	**EHR**	**95% CI**
*1 year*								
1979–1982	1.00	—	1.00	—	1.00	—	1.00	—
1983–1986	0.85	0.69–1.05	0.95	0.66–1.36	1.05	0.79–1.39	0.99	0.81–1.22
1987–1990	0.74	0.60–0.91	0.67	0.45–0.99	0.79	0.59–1.07	0.79	0.65–0.98
*Trend*^e^	*0.86*	*0.78*–*0.95*	*0.85*	*0.70*–*1.03*	*0.89*	*0.77*–*1.03*	*0.89*	*0.81*–*0.99*
								
*5 years*								
1979–1982	1.00		1.00		1.00		1.00	
1983–1986	1.05	0.88–1.25	0.97	0.73–1.28	1.03	0.83–1.27	1.16	0.97–1.39
1987–1990	0.86	0.72–1.02	0.74	0.55–1.00	0.88	0.70–1.11	1.13	0.96–1.34
*Trend*	*0.90*	*0.83*–*0.99*	*0.88*	*0.75*–*1.02*	*0.92*	*0.82*–*1.03*	*0.91*	*0.83*–*0.99*

Excess hazard ratios estimated separately for each subsite. Final model for each subsite includes only those covariates that significantly improved the goodness of fit (see text).

Data from 1979–1990 only because jejunal tumours were not separately identifiable before 1979.

a Covariates age, deprivation, morphology.

b Covariates deprivation, morphology.

c Covariates age, morphology.

d Covariates age, deprivation, region, morphology.

e Ratio of the excess hazard of death between successive calendar periods of diagnosis, after adjustment for covariates that significantly improved the goodness of fit.
